# Supplementary material for: Transposable elements generate regulatory novelty in a tissue-specific fashion
Source: BMC Genomics. 2018 Jun 18;19:468. doi: 10.1186/s12864-018-4850-3 (PMC6006921; doi:10.1186/s12864-018-4850-3)
Supplement: Supplementary file 9 — It contains five “HTML” files with the following names: HERV15_meme-chip.html; LTR13_PANCREAS_meme-chip.html; SVA_ADIPOSE_NUCLEI_meme-chip.html; SVA_LIVER_meme-chip.html; X7C_CHarlie15a_BREAST_ACTIVE.html. The HTML files represent the outputs of the “MEME-ChIP” analyses (i.e. motif analyses) for: HERV15 regions in the liver; LTR13C_ in the pancreas; SVAs in adipose nuclei and liver; X7C_Charlie_15a in Breast. (ZIP 399 kb) [file 12864_2018_4850_MOESM9_ESM.zip › Supplemental_File_S2_LIGHT/HERV15_meme-chip.html]

MEME ChIP


This is a link to a summary of the MEME-ChIP results in an easy-to-parse
"tab-separated values" format. Each line gives values for one motif found by MEME-ChIP.
The fields are:

- MOTIF\_INDEX -- The index of the motif in the "Motifs in MEME text format" file.- MOTIF\_SOURCE -- The name of the program that found the *de novo* motif, or the
    name of the motif file containing the known motif.- MOTIF\_ID -- The primary ID of the motif.- ALT\_ID -- The alternate ID of the motif.- CONSENSUS -- The ID of the *de novo* motif, or a consensus sequence
          computed from the letter frequencies in the known motif (see below).- WIDTH -- The width of the motif.- SITES -- The number of sites reported by the *de novo* program, or the number
              of "Total Matches" reported by CentriMo.- E-VALUE -- The statistical significance of the motif.- E-VALUE\_SOURCE -- The program that reported the *E*-value.- MOST\_SIMILAR\_MOTIF -- The known motif most similar to this motif according to Tomtom.- URL -- A link to a description of the most similar motif or to the known motif.

[
close ]

This is a link to a file containing all the significant motifs found by MEME-ChIP.
The motifs are in MEME text format, and their IDs correspond to the
motif indices given in the "Summary in TSV Format" file.
**Note:**The "nsites=" and "E=" fields in the motif headers are only
relevant for the MEME and DREME motifs. For known motifs, those values do
not refer to the number of sites in the input sequences.

[
close ]

This is a link to the motif in the output of the particular motif
discovery (e.g., MEME) or motif enrichment (e.g., CentriMo) program that
reported it.

[
close ]

This is the significance of the motif according to the particular motif
discovery (e.g., MEME) or motif enrichment (e.g., CentriMo) program that
reported it.

Follow the link under the "Discovery/Enrichment Program" column for
more information on how the significance value was derived.

[
close ]

Motifs reported by a motif discovery program (e.g., MEME) are compared
with known motifs in a motif database specified by the user. This column
lists the (up to) three most similar motifs. Only known motifs with
TOMTOM similarity E-values of less than 1.0 to the discovered motif will
be shown here. Clicking any of these links will show the TOMTOM results
where all alignments can be viewed.

Motifs reported by a motif enrichment program (e.g., CentriMo) list the
motif's name and a link to the motif's entry on the database website if it
is available.

[
close ]

This graph shows the distribution of the best matches to the motif in
the sequences as found by a CentriMo analysis.

The vertical line in the center of the graph corresponds to the center
of the sequences.

Clicking on a motif's graph will take you to the CentriMo output with
that motif selected for graphing.

[
close ]

Clicking here will show you all the motifs found by motif discovery or
motif enrichment analysis that are significantly similar to the reported
motif.

The additional motifs are shown aligned with the reported motif,
sorted in order of significance of the motif according to the
particular motif discovery (e.g., MEME) or motif enrichment
(e.g., CentriMo) program that reported it.

To cluster the motifs MEME ChIP does the following:

1. Start with no groups and all significant reported motifs.
2. Run TOMTOM with all significant reported motifs to determine
   pairwise similarity.
3. Group Highly Similar Motifs---

   While ungrouped motifs:

   Select most significant ungrouped motif.

   This is called the "seed" motif for the group and we will call the
   E-value of its seed motif the group's "significance".

   Form a new group from the seed motif and all other motifs that
   are not yet in a group and who are strongly similar to the seed
   motif (default: TOMTOM E-value ≤ 0.05).
4. Merge Groups---

   For each group (most significant to least significant), merge it with
   any less significant group if all of its motifs are weakly similar to
   the first group's seed motif (default: TOMTOM E-value ≤ 0.1).

[
close ]

Clicking here takes you to the CentriMo motif enrichment analysis with
the results for this all the motifs in this group.

[
close ]

This lists links to related content, which may include:

- Motif Spacing Analysis--SpaMo results using this motif as
  the "primary" motif, and each of the discovered motifs and
  motifs in any motif databases specified to MEME-ChIP as
  potential "secondary" motifs. SpaMo reports the secondary
  motifs whose occurrences are enriched at particular distances relative
  to the primary motif's occurrences in the input sequences.- Motif Sites in GFF3--FIMO results showing the positions of occurrences
    of this motif in the input sequences in GFF3
    format. If the input sequences to MEME-ChIP have FASTA headers following
    the UCSC style ("chromosome\_name:starting\_position-ending\_position"),
    and the chromosome names are in UCSC (not ENSEMBL) format,
    the GFF3 output will be suitable for uploading to the UCSC Genome Browser
    as a custom track.

[
close ]

# MEME-ChIP

## Motif Analysis of Large Nucleotide Datasets

If you use MEME-ChIP in your research, please cite the following paper:  

Philip Machanick and Timothy L. Bailey, "MEME-ChIP: motif analysis of large DNA datasets",
*Bioinformatics*, **27**12, 1696-1697, 2011.
[full text]

Motifs
  |  
Programs
  |  
Input Files
  |  
Program information
  |  
Summary in TSV Format  
  |  
Motifs in MEME Text Format


# Javascript is required to view these results!

# Your browser does not support canvas!


## Motifs

The significant motifs
(E-value ≤ )
found by the programs MEME, DREME and CentriMo;
clustered by similarity and ordered by E-value.

Expand All Clusters
Collapse All Clusters

## Programs

## Input Files

#### Motifs

##### MEME-ChIP version

(Release date: )

##### Reference

Philip Machanick and Timothy L. Bailey, "MEME-ChIP: motif analysis of large DNA datasets",
*Bioinformatics*, **27**12, 1696-1697, 2011.

##### Command line summary
